# Diagnosis, treatment, and management of mediastinal masses

**DOI:** 10.1590/1806-9282.20250440

**Published:** 2025-10-27

**Authors:** Kubra Nur Kilic, Omer Topaloglu, Sami Karapolat, Atila Turkyilmaz, Ali Akdogan, Celal Tekinbas

**Affiliations:** 1 Karadeniz Technical University, Faculty of Medicine, Department of Thoracic Surgery – Trabzon, Turkey.; 2 Recep Tayyip Erdogan University, Faculty of Medicine, Department of Thoracic Surgery – Rize, Turkey.; 3 Karadeniz Technical University, Faculty of Medicine, Department of Anesthesiology – Trabzon, Turkey.

**Keywords:** Sternotomy, Mediastinum neoplasms, Median sternotomy, Video assisted thoracoscopic surgery, VATS

## Abstract

**OBJECTIVE:**

Mediastinal neoplasms are relatively rare tumors that require surgical or non-surgical treatment, and they harbor a wide variety of cell types. The aim of this study was to evaluate the surgical outcomes and prognosis of patients who underwent surgery for mediastinal masses.

**METHODS:**

The age, gender, symptoms, comorbid factors, radiological findings, preoperative diagnostic steps, surgical procedure, mortality, complications, length of hospital stay, histopathological diagnosis, and survival of 118 patients with mediastinal masses operated on for diagnosis and treatment between January 2013 and December 2018 were retrospectively analyzed.

**RESULTS:**

When the mediastinal masses were evaluated according to compartments, the most common mass was found in the anterior mediastinum (n=72, 61%). While 83.3% of malignant lesions were located in the anterior mediastinum, no malignant lesion was detected in the posterior mediastinum. There was a significant correlation between the compartment where the mass was located and its malignancy. A significant increase in the malignancy rate was observed among male patients. The likelihood of malignancy increased with increasing tumor size (p=0.002). Complications were shown to significantly extend the length of hospitalization (p=0.018). Overall survival was found to be at an average of 71.46±2.1 months. The 5-year survival rate was found to be 88.1%. There was a significant difference in survival between genders, in favor of the female sex (p=0.02).

**CONCLUSION:**

Considering the low morbidity and mortality rates of mediastinal masses, surgical intervention should be performed promptly for the diagnosis and treatment of mediastinal masses. With videothoracoscopy, adequate biopsies can be obtained from unresectable masses and complete excision of benign lesions can be achieved safely.

## INTRODUCTION

The mediastinum extends from the sternum anteriorly to the vertebral column posteriorly, and from the thoracic inlet superiorly to the diaphragm inferiorly. Located in the center of the thoracic cavity, it is divided into anterior, middle, and posterior compartments. Masses originating from the structures in the compartments vary, and each cyst or tumor has a tendency to form in its own specific compartment^1^.

Mediastinal neoplasms are relatively rare tumors that require surgical or non-surgical treatment and harbor a wide variety of cell types^2^. They may be benign or malignant in behavior, solid or cystic in nature, and may involve any compartment in the mediastinum^1^. They can be seen in a wide age range, although they are more common between 30 and 50 years of age. The type of mediastinal tumor is usually related to the age of the patient. In children, the compartment associated with mediastinal masses is usually the posterior mediastinum, and the tumor is more likely to be benign, whereas in adults, the anterior mediastinum is the most associated compartment, and tumors tend to be malignant in most cases^3,4^. As the tumor grows and progresses to adjacent mediastinal regions, the localization and relationship of tumors may be disrupted. For example, a germ cell tumor originating from the anterior mediastinum may extend to the vertebrae, or a non-Hodgkin's lymphoma (NHL) may involve the entire mediastinum^5^. This is more commonly observed in children and in cases with larger tumors, and should be taken into consideration^6^.

Adequate knowledge of the radiologic features of mediastinal masses facilitates the definitive diagnosis and differentiation of these masses^7^. Different approaches have been adopted to obtain the type and pathology of mediastinal masses based on the location of the mass. Radiology-guided needle biopsy, endoscopic needle biopsy, anterior mediastinotomy, video-assisted thoracic surgery (VATS), and open surgical approaches such as sternotomy and thoracotomy are among the methods that can be used to obtain tissue diagnosis^8^.

In this study, we aimed to evaluate the surgical outcomes in patients who underwent mediastinal mass excision with various operative procedures due to mediastinal masses.

## METHODS

### Patients and study protocol

The age, gender, symptoms, comorbid factors, radiological findings, preoperative diagnosis, surgical procedure, mortality, complications, length of hospital stay, histopathological diagnosis, and survival of 118 patients with mediastinal masses who were operated on for diagnosis and treatment between January 2013 and December 2018 were retrospectively analyzed by scanning the archive files.

Esophageal pathologies, secondary mediastinal masses, and lung malignancies were excluded. Postoperative follow-up of the patients was performed through outpatient clinic visits in the first 1-year period. Survival and death dates of the patients were obtained from the hospital data system. Factors that may affect mortality, morbidity, and survival were evaluated according to histopathologic diagnosis.

### Statistical analysis

Survival probabilities were calculated using the Kaplan-Meier method, and survival comparisons were conducted using the log-rank test. A significance level of p<0.05 was adopted for all statistical analyses.

## RESULTS

The most common diagnosis was thymic lesions with 41 cases (34.8%). The histopathologic distribution of the cases is shown in Table 1.

**Table 1:** Histopathologic distribution of cases.

Histopathologic type	n	%
**Thymic lesions**	**41**	**34.8**
Thymoma*	27	22.9
Thymic lymphoid hyperplasia	6	5.2
Thymus tissue	3	2.5
Thymolipoma	2	1.7
Thymic carcinoma	3	2.5
**Benign cystic lesions**	**21**	**18.3**
Pericardial cyst	10	8.7
Bronchogenic cyst**	5	4.3
Pleural cyst	3	2.5
Esophageal cyst	1	0.8
Thoracic duct cyst	1	0.8
Hydatid cyst	1	0.8
**Lymphoma**	**18**	**15.6**
Non-Hodgkin's lymphoma	12	10.4
Hodgkin's lymphoma	6	5.2
**Thyroid lesions**	**15**	**12.8**
Nodular colloidal goiter	5	4.3
Thyroid papillary carcinoma	3	2.5
Follicular nodular disease	3	2.5
Hashimoto's thyroiditis	2	1.7
Ectopic thyroid	1	0.8
**Neurogenic tumors**	**7**	**6**
Schwannoma	6	5.2
Paraganglioma	1	0.8
**Germ cell tumor**	**4**	**3.4**
**Mesenchymal tumors**	**3**	**2.5**
Lipoma	1	0.8
Solitary fibrous tumor	1	0.8
Malignant mesenchymal tumor	1	0.8
**Hamartoma**	**2**	**1.7**
**Parathyroid adenoma**	**2**	**1.7**
**Reactive lymphadenopathy**	**2**	**1.7**
**Tumor necrosis**	**1**	**0.8**
**Thymoma—lymphoma could not be differentiated**	**1**	**0.8**
**Change secondary to treatment**	**1**	**0.8**
**Total**	**118**	**100**

* One case of thymoma had a concomitant thymic cyst, and

** one case of bronchogenic cyst had an associated esophageal cyst. The second column of the bold values represents the number of patients (n) and the third column represents the percentage (%).

Classification of mediastinal masses according to compartments and mass types is shown in Table 2.

**Table 2:** Comparison of malignant and benign lesions in compartments.

Type Compartment	Benign (59.3%)	Malignant (40.7%)	Total (n)
	n (%)	n (%)	
Anterior	32 (45.7)	40 (83.3)	72
Middle	28 (40)	8 (16.7)	36
Posterior	10 (14.3)		10

There was a significant correlation between the compartment where the mass was located and malignancy (p<0.001).

When the gender–malignancy relationship was analyzed, it was observed that the malignancy rate was significantly higher in the male sex compared to the female sex (p=0.007).

Notably, 72% of the cases were symptomatic at the time of diagnosis. The mean tumor size calculated according to imaging methods and pathological material was 9.8 cm in malignant lesions and 6.6 cm in benign lesions. There was a statistically significant correlation between tumor size and malignancy (p=0.002).

When the tumor diameter was compared according to the surgical approach, the mean tumor diameter was 4.79±2.9 cm (1.5–13 cm) in VATS patients, 7.1±3.2 cm (2–17 cm) in thoracotomy patients, and 9.2±4.6 cm (1–15 cm) in sternotomy patients (p<0.001).

The mean length of hospitalization was 5.27±3.38 days (min: 1, max: 21 days). Minimally invasive surgical approach resulted in shorter postoperative hospitalization compared to open surgery. While the duration of hospitalization was significantly shorter in VATS patients (p<0.001), there was no significant correlation between the presence of comorbidities and the duration of hospitalization (p=0.551).

The most common postoperative complications are shown in Table 3. The average length of hospitalization without complications was 4.7 days, whereas the average length of hospitalization when complications occurred was 7.03 days. Complications had a significant effect on the length of hospitalization (p=0.018).

**Table 3:** Number of postoperative complications.

Complications	Number (n)
Pneumothorax	3
Diaphragm elevation	3
Atelectasis	2
Pneumonia	2
Horner's syndrome	2
Wound site infection	2
Atrial fibrillation	2
Postoperative intubation	2

### Survival

The patients were followed up for a period ranging from 0 to 80 months, with a mean follow-up duration of 42.80±22.47 months. During this follow-up period, 14 patients (11.86%) died. The mean overall survival was 71.46±2.1 months, and the 5-year survival rate was 88.1%.

There was a significant difference in survival between sexes (p=0.02). The mean survival was 76.2 months (95%CI 72.6–79.8 months) for women and 62.8 months (95%CI 53.5–72.1 months) for men.

The mean survival for cases of malignant mediastinal masses was 59.4 months (95%CI 50.3–68.5 months), with a 5-year survival rate of 70.8%. While there was no significant difference between the presence of complications and survival, age significantly affected survival (p<0.001).

Although the type of resection (biopsy vs. excision) did not significantly affect survival in malignant cases (p=0.07), the mean survival was 67.5 months (95%CI 66.4–78.5 months) for cases with total mass excision and 51.1 months (95%CI 37.4–64.7 months) for cases with partial excision or biopsy.

## DISCUSSION

This study highlights five key points regarding the approach and management of mediastinal masses: (a) the incidence of mediastinal masses has recently increased; (b) male sex, anterior mediastinal location, and larger tumor size are prognostic factors for malignancy; (c) anterior mediastinotomy should be preferred for the definitive diagnosis of anterior mediastinal masses; (d) VATS is the most useful surgical approach, particularly for the excision of benign lesions in the middle and posterior mediastinum; and (e) the incidence of intrathoracic thyroid lesions was significantly higher in our study compared to the literature.

Although the incidence of primary mediastinal masses varies across different series, it is generally low. For instance, Davis et al.^9^ reported 400 cases over 56 years, Benjamin et al.^10^ reported 215 cases over 20 years, Rubush et al.^11^ reported 186 cases over 20 years, and Ovrum et al.^12^ reported 91 cases over 20 years. In our study, the finding of 118 cases of primary mediastinal masses in just 5 years is remarkable. This increase is clearly related to more frequent and earlier diagnoses, which can be attributed to recent advancements in radiological technology, enhanced screening programs, and the rising number of well-equipped hospitals.

Strollo et al.^13^ reported in a 1997 literature review that two-thirds of mediastinal lesions were benign, and similarly, benign lesions constituted 59.3% of cases in our series. In our study, benign lesions were observed more frequently in females, while the malignant nature of mediastinal lesions in males was significant.

Approximately half of the primary mediastinal lesions are located in the anterior mediastinum. This is followed by the posterior and middle mediastinum. Notably, 59% of masses in the anterior mediastinum, 29% in the middle, and 16% in the posterior mediastinum are malignant^2^. In our study, the most common lesion was found in the anterior mediastinum: 85.9% of malignant lesions were in the anterior mediastinum. While 22.2% of the lesions in the middle mediastinum were malignant, no malignant lesion was found in the posterior mediastinum.

Cohen et al.^14^ associated the presence of symptoms, location of the lesion in the anterior mediastinum, and tumor size with the presence of malignancy. In our study, although larger tumor size, male sex, and anterior mediastinal location were found to be significant in favor of malignancy, no correlation was found between the presence of symptoms and malignancy.

Tissue diagnosis is very important, especially in anterior mediastinal masses, as it is a guide for treatment modification. The most common invasive diagnostic procedure we performed preoperatively was computed tomography (CT)-guided fine-needle aspiration biopsy (FNAB). While Assaad et al.^15^ could not definitively diagnose 18% of cases in their series of 157 patients who underwent CT-guided IAB, Petranovic et al.^16^ reported a rate of 23% in their series of 52 patients and Blegvad et al.^17^ reported 52% in their series of 87 patients. In our series of 18 patients, this rate was 44%. While we were able to make a definitive diagnosis in 100% of the cases with anterior mediastinotomy, it was remarkable that this rate remained at 56% with IAB, necessitating the use of additional methods. We believe that anterior mediastinotomy should be preferred as the diagnostic procedure for anterior mediastinal masses since resorting to additional methods to make a definitive diagnosis is time-consuming and more costly.

In the past, resection through median sternotomy without biopsy was generally recommended for clinically benign and resectable mediastinal masses, but with recent advances in thoracoscopic methods, VATS has now surpassed open surgical procedures with less morbidity^18^. With VATS, all mediastinal compartments can be accessed with equal reliability and efficiency, and a panoramic view of the mediastinal mass can be obtained^19^. In our study, VATS was performed in 25 patients, including six biopsies and 19 excisions, and all lesions were in the middle and posterior mediastinum. All lesions excised by VATS were benign. While there was no significant difference in complication rates compared to open surgical methods, it was analyzed that the length of hospital stay was significantly reduced, as in the series of Akashi et al.^19^ With thoracoscopy, both adequate biopsies can be obtained from unresectable lesions and benign lesions can be safely resected^20^. For these reasons, the VATS method is nowadays more preferred because of its superiority over open surgery, i.e., thoracotomy. Consistent with this, our study reflects the frequent use of VATS in daily clinical practice. Approximately 21% of the patients in our study were operated on with the VATS approach. The usage of VATS was less common in our study compared to open surgery, which reflects the recent introduction of VATS at the start of the study period and the time required to complete the learning curve for this technique. We attribute the relatively lower VATS rates in our study to these reasons. However, we would like to state that these rates have improved considerably in today's conditions and have reached 80%. We believe that VATS is the most effective surgical method, especially in the resection of benign lesions in the middle and posterior mediastinum, as it provides less morbidity and shorter hospital stay.

Intrathoracic thyroid lesions constitute 5.8% of mediastinal masses^21^. Although centers worldwide performing a large number of thyroid surgeries have reported that the incidence of retrosternal goiter varies between 3 and 30%, Monchik et al.^22^ showed in their series of 976 patients undergoing thyroid surgery that CT and operative findings showed extension to the thoracic inlet, suggesting a mediastinal mass in at least 50% of cases^23^. There is a general consensus that the majority of mediastinal goiters can be successfully excised through a cervical approach and that thoracic intervention is rarely necessary. The need for sternotomy in the excision of these goiters varies between 0 and 40%^23^. Cohen et al.^24^ excised 108 of 113 cases of mediastinal goiter, Wright et al.^18^ excised 75 of 97 cases, and Nakaya et al.^25^ excised all 44 cases by transcervical approach. In our study, all cases of mediastinal thyroid lesions required a thoracic approach. Although the majority of goiter cases were operated on by Otorhinolaryngology and General Surgery clinics, the rate of mediastinal thyroid lesions in our series was 12.7%, which is considerably higher than reported in the literature. This is obviously due to the fact that goiter is an endemic disease in our region.

The limitations of our study include its retrospective nature, single-center design, long study period, and the inclusion of a limited number of patients.

## CONCLUSION

Despite its small size, the mediastinum is an important anatomical region housing a wide array of tumors with diverse types and characteristics. It is evident that there is an increasing trend toward earlier and more frequent diagnoses, facilitated by recent advancements in radiological technology. Advanced age and male sex have been identified as independent factors that decrease survival in malignant mediastinal masses. Considering the low mortality rates for the diagnosis and treatment of mediastinal masses, surgical intervention should be performed promptly in most cases. The prognosis is typically very favorable in early-stage thymomas that are completely resected. In order to increase survival, all thymoma cases should be considered malignant, and aggressive surgery should be more encouraged to achieve complete resection in these cases. It should be kept in mind that the incidence of mediastinal thyroid lesions may increase according to the conditions of the region. VATS is considered the standard treatment for mediastinal cysts due to its low morbidity and mortality rates, quick recovery, and significantly reduced hospital stay compared to conservative methods. With thoracoscopy, adequate biopsies can be obtained from unresectable masses and complete excision of benign lesions can be achieved safely. We believe that VATS is the most useful surgical method for resection of benign lesions, especially in the middle and posterior mediastinum, as it provides less morbidity and shorter hospital stay.

## Data Availability

The datasets generated and/or analyzed during the current study are available from the corresponding author upon reasonable request.
